# Discovery of moiety preference by Shapley value in protein kinase family using random forest models

**DOI:** 10.1186/s12859-022-04663-5

**Published:** 2022-04-15

**Authors:** Yu-Wei Huang, Yen-Chao Hsu, Yi-Hsuan Chuang, Yun-Ti Chen, Xiang-Yu Lin, You-Wei Fan, Nikhil Pathak, Jinn-Moon Yang

**Affiliations:** 1 Institute of Biomedical Engineering, National Yang Ming Chiao Tung University, Hsinchu, Taiwan; 2 Institute of Bioinformatics and Systems Biology, National Yang Ming Chiao Tung University, Hsinchu, Taiwan; 3 Institute of Molecular Medicine and Bioengineering, National Yang Ming Chiao Tung University, Hsinchu, Taiwan

**Keywords:** Kinase family inhibitor, Random forest, Shapley Additive exPlanations (SHAP) approach, Merged moiety-based interpretable features (MMIFs), Kinase inhibitors

## Abstract

**Background:**

Human protein kinases play important roles in cancers, are highly co-regulated by kinase families rather than a single kinase, and complementarily regulate signaling pathways. Even though there are > 100,000 protein kinase inhibitors, only 67 kinase drugs are currently approved by the Food and Drug Administration (FDA).

**Results:**

In this study, we used “merged moiety-based interpretable features (MMIFs),” which merged four moiety-based compound features, including Checkmol fingerprint, PubChem fingerprint, rings in drugs, and in-house moieties as the input features for building random forest (RF) models. By using > 200,000 bioactivity test data, we classified inhibitors as kinase family inhibitors or non-inhibitors in the machine learning. The results showed that our RF models achieved good accuracy (> 0.8) for the 10 kinase families. In addition, we found kinase common and specific moieties across families using the Shapley Additive exPlanations (SHAP) approach. We also verified our results using protein kinase complex structures containing important interactions of the hinges, DFGs, or P-loops in the ATP pocket of active sites.

**Conclusions:**

In summary, we not only constructed highly accurate prediction models for predicting inhibitors of kinase families but also discovered common and specific inhibitor moieties between different kinase families, providing new opportunities for designing protein kinase inhibitors.

## Introduction

Human cancer, immune diseases, and complex diseases are related to protein kinase signaling pathways; therefore, protein kinases have become the second-largest drug target family [1]. There are > 100,000 recorded protein kinase inhibitors, however only 67 small molecule kinase drugs have been approved by the Food and Drug Administration (FDA) [2]. The highly conserved ATP-binding pocket of kinases is a major limitation for development of drug-resistance [3]. During the progression of drug resistance, diseases are regulated by kinase families rather than by a single kinase, which is in line with our previous research [4]. Another study also indicated that three protein kinases in the JAK family co-regulate and transmit signals in the biological pathways of inflammation and immune regulation. Therefore, there is a strategy to treat complex diseases by developing inhibitors of the protein kinase family. Some FDA-approved drugs are kinase family inhibitors, such as baricitinib, a JAK1 and JAK2 inhibitor, which stop the growth of cancer cells, thus reducing downstream immune cell function [5, 6]. However, the experimental analysis of large compounds on protein kinases is time-consuming and costly [7]. Currently, machine learning methods provide a faster method for drug development. Some studies have developed classifiers of inhibitors and non-inhibitors of a single protein with chemical and biological descriptors as input features. Benjamin et al. constructed ~ 200 kinase inhibitor prediction models using a random forest. DEEPScreen used deep convolutional neural networks to train the 704 proteins prediction model, and Minjian et al*.* developed a JAK2 kinase inhibitor prediction model for the treatment of myeloproliferative neoplasm [8–10]. However, there is a lack of kinase family inhibitor prediction models and strategies to open the black box of machine learning.

In this study, we identified kinase family inhibitors by kinase profiling and using the ChEMBL database. We proposed a random forest (RF) model to predict protein kinase family inhibitors that utilize moiety-based interpretable features (MMIFs) as input features. The 10 kinase family prediction models were independently constructed. Furthermore, we aim to open the black box of the model and explain the prediction result of the model using the Shapley Additive exPlanations (SHAP) methodology [11]. Moreover, we identified common and specific moieties, which are important moieties interacting with key motifs of kinases, such as hinge, DFGs, or P-loops in the ATP pockets, across kinase families, and verified the results by protein kinase-inhibitor structure complexes.

## Results

**Fig. 1:**
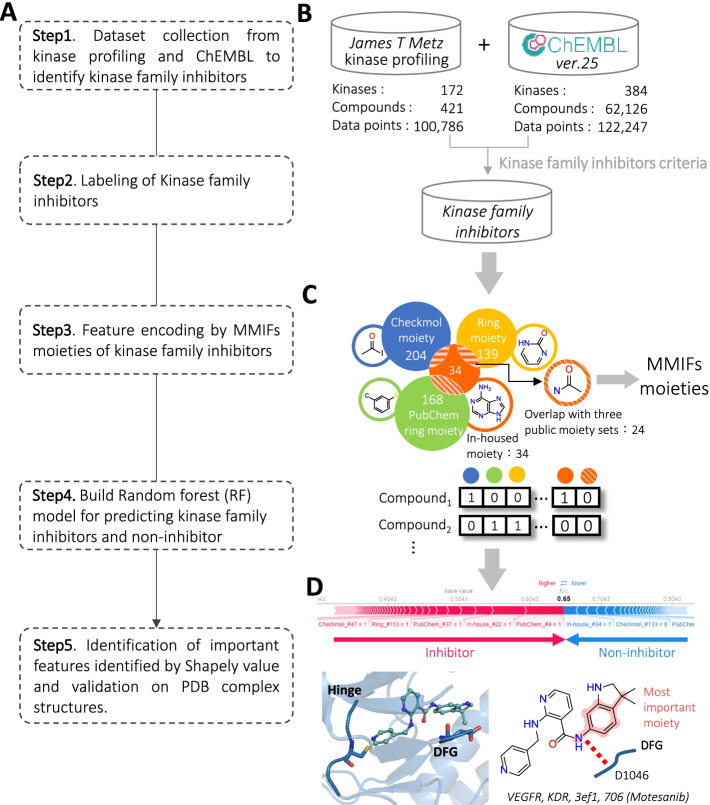
Overview of establishing kinase family inhibitor model and identifying key features. **A** Main procedure. **B** Dataset collection and identification of kinase family inhibitors. **C** Feature encoding with the MMIFs, which combined the moieties of Checkmol, ring moiety, PubChem, and in-housed moieties. **D** Identification of important moieties by Shapley value and validation by kinase-inhibitor structure complexes

Figure 1 shows the major steps for establishing a kinase family inhibitor model and identifying the workflow of the key features. We approached kinase family inhibitor prediction as a binary classification problem, with each of them treated as an individual predictor for a target protein kinase family. First, we collected kinase-inhibitor data from the kinase profiling of Metz et al. [12] and ChEMBL version 25 [13]. Then, we defined the inhibitor sets for kinase families. Second, we represented compounds in the form of 518 descriptors, which combined Checkmol moieties [14], ring section of PubChem fingerprints, rings in drugs [15], and in-housed predefined moieties from metabolites and approved small molecular drugs. Next, we utilized 518 binary descriptors and built RF models for kinase family inhibitor prediction. Finally, to interpret the black box, we used the Shapley value to reveal the contribution of each moiety and validate the significance of moieties with protein–ligand complexes from protein data bank (PDB).

### Features comparation and model performance

**Fig. 2:**
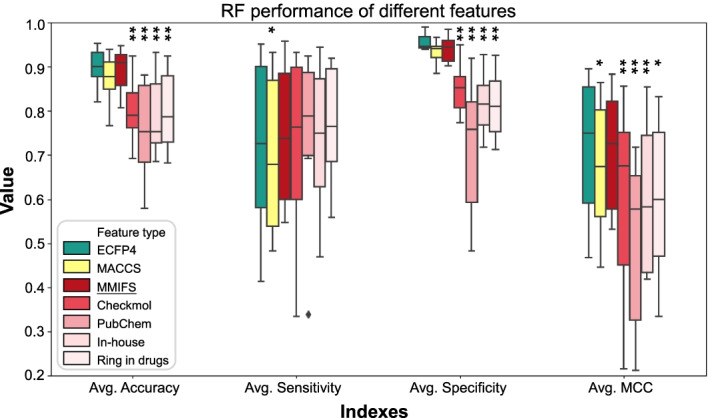
Boxplots of Random Forest (RF) model quality metrics and comparison of different features. The performance indexes were the average of 50 RF models. The result indicated that MMIFs show significant improvement compared to the separated features (e.g. Checkmol, PubChem, in-housed, and ring in drugs). Although models based on MMIFs show slightly lower results compared to the model with ECFP4, MMIFs are able to explain the model results with the substructure features on compounds. Statistical analysis was performed by student T-test with *p* values compared to the MMIFs. Single stars denote 0.01 < *p* < 0.05, double stars 0.001 < *p* < 0.01

In order to evaluate whether MMIFs improve the model performances compared to the features separately (e.g., Checkmol, PubChem, in-housed, and ring in drugs), we built an RF model with different features. Moreover, to further assess the quality of the models with MMIFs, we also built models of ECFP4 and MACCS. The boxplot results were based on the testing results of the average of 10 kinase families with 50 prediction models (Fig. 2). The RF models yielded reasonable prediction results compared to the MMF features with an average accuracy of 0.85 ± 0.12, a sensitivity of 0.76 ± 0.23, a specificity of 0.93 ± 0.05, and an MCC of 0.72 ± 0.21. Notably, the models with MMIFs show a significant improvement compared to that with separated features (e.g., Checkmol, PubChem, in-housed, and ring in drugs) in most of the performance indexes. On average, models with MMIFs showed an improvement of approximately 10% in accuracy, 15% in specificity, and 10% in MCC. In addition, the performance improvements of MMIFs over that of separated features are statistically significant (for example p values of accuracy for MMIFs vs Checkmol: 0.001; MMIFs vs PubChem: 0.0003; MMIFs vs In-house: 0.0005; MMIFs vs Ring in drugs: 0.004). Despite the fact that when comparing models with other features, such as ECFP4 and MACCS, which are commonly used in machine learning for representing compounds, the performance of models with MMIFs is slightly lower than that of models with ECFP4. However, the features of ECFP4 cannot illustrate and compare the same or different kinase families by the same moiety because the features were generated by each compound surrounding the environment. On the other hand, predefined moiety features could explain and compare with the same or different families and indicate common or specific moiety preferences. In summary, models with MMIFs show the best performance compared to that of models with predefined substructure moieties and have the ability to interpret the black-box machine learning in the model.

### 2.2. Interpretation of model by SHAP

**Fig. 3:**
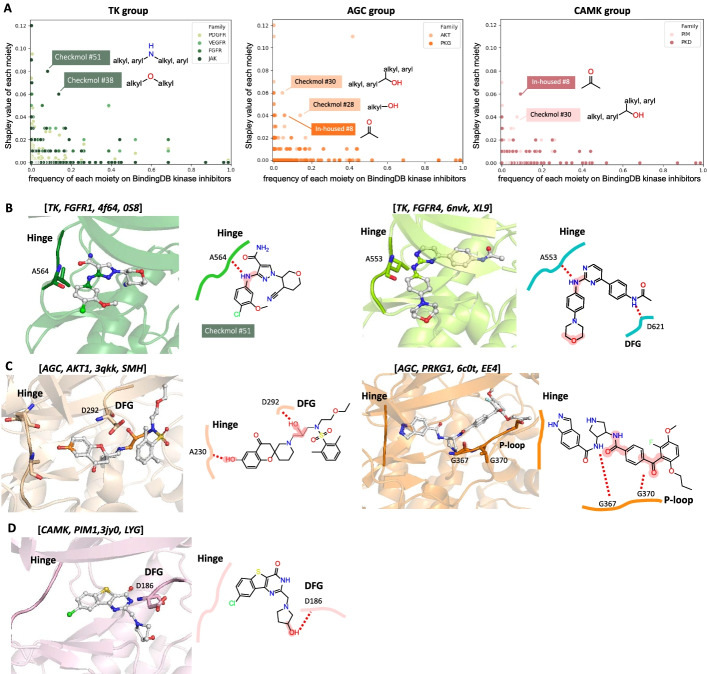
Validation of important moieties identified by Shapley value. **A** Scatter plot of Shapley value and the occurrence ratio of kinase inhibitors for each moiety. The indicated features are with high Shapley value and low occurrence ratio. Example of important moieties validated in the protein kinase inhibitor complexes of (**B**) TK group, FGFR1, and FGFR4 in the FGFR family, **C** AGC group, AKT1, and PRKG1 in the PKG kinase family, and **D** CAMK group and PIM1 in the PIM kinase family. The label title of **B**–**D** indicate [*kinase group*, *kinase family*, *PDB code*, *Ligand ID*]

In this study, we utilized SHAP to identify important features and to open the black box of the RF model. However, the question is aroused whether SHAP prefers to identify the high occurrences moieties and therefore not the exact important moieties. To test whether the SHAP is meant to indicate the important features of the inhibitors, we collected nearly 100,000 kinase inhibitors from the BindingDB database [16]. We calculated the frequency of 518 MMIFs moieties occurring in over 100,000 kinase inhibitors and compared it with the Shapley value of each moiety. The scatter plot results of the TK, AGC, and CAMK groups are shown in Fig. 3A. Surprisingly, we found that the SHAP of each moiety is not correlated to the frequency of the moiety (i.e., the moieties with high Shapley value do not contribute to the moiety with high frequency). We further examined the importance of the moieties with high SHAP (top 30 in each family) and low frequency (less than 0.2) that appeared on kinase inhibitors, which were identified in the TK (e.g., Checkmol #51, and Checkmol #38), AGC (e.g., Checkmol #30, Checkmol #28, and in-housed #8), and CAMK group (e.g., in-housed #8 and Checkmol #30). To further verify the moieties with the roles in structure complexes, we collected the complexes in PDB with the following criteria: (a) protein with Pfam ID of kinase domain, PF00069 (Pkinase), PF07714 (Pkinase_Tyr), and PF00433 (Pkinase_C, Pkinase) and (b) structures with ligand complexed. In total, 3,569 kinase-inhibitor complexes were collected and for further investigation.

Moreover, we found that the inhibitors, 0S8 and XL9, are complexes with the FGFR family with Checkmol #51 and Checkmol #38, respectively. Both of the Checkmol #51 moiety form the hydrogen bond with crucial residues on the hinge motif of the kinase, A584 of FGFR1 and A553 of FGFR4, respectively. Also, Checkmol fingerprint #38 interacts with D621 on the DFG motif of FGFR4 (Fig. 3B). Similarly, Fig. 3C shows the AGC family complexed with ligands SMH and EE4, that with Checkmol #28 and Checkmol #30, which are have high SHAP but the low frequency, forming key interactions to the important motifs, such as DFG motif and P-loop. The same observation on the PIM family is shown in Fig. 3D. In conclusion, the moieties with high Shapley values are not dependent on the frequency of occurrence and are important in interacting with key motifs of protein kinases, such as hinge, DFG, and P-loop.

### Identification of common and specific moieties across kinase families

**Fig. 4:**
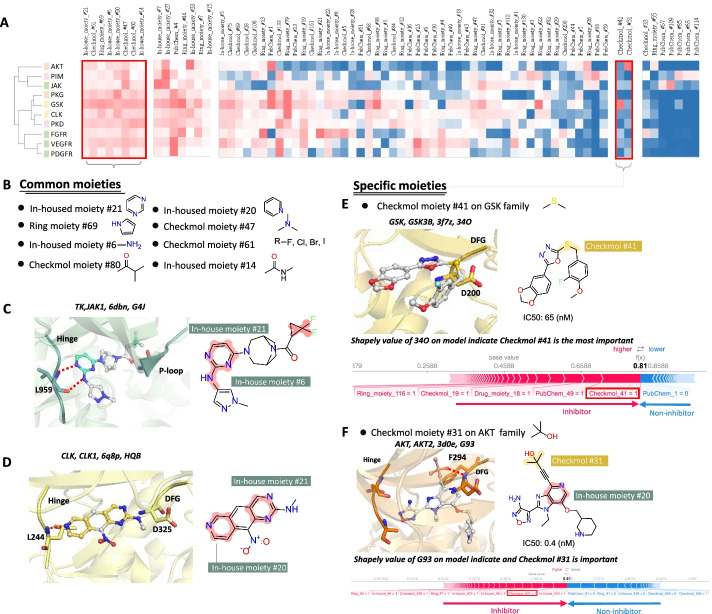
Identification of common and specific moieties of 10 kinase families. **A** Hierarchical clustering of top 30 Shapely value (SHAP) moieties on 10 kinase families and the identified common/specific moieties. **B** List of 8 common moieties across 10 kinase families. Example of common moieties of (**C**) the JAK kinase family validated by complex structure, which interact with hinge and p-loop, and **D** on the CLK kinase family, which interact with hinge and DFG. **E** Specific moieties, Checkmol moiety #41, on the GSK family was identified and validated by the contribution of the Shapley value. **F** Specific moieties, Checkmol moiety #31, on the AKT family was identified and validated by the contribution of the Shapley value

We first investigated the SHAP and proposed that the value shows biological value. We then further asked whether these families have common or specific moieties, which is crucial for further kinase inhibitor design. Hierarchical clustering was performed with the top 30 SHAP moieties of 10 kinase families (Fig. 4A). We further identified eight common moieties with high SHAP for each kinase family and validated the common moieties by the complex structures (Fig. 4B). Surprisingly, four out of eight common moieties were in-housed moieties and formed a key interaction on the structure complexes. For example, in the Jak1 complex with inhibitor G4J (PDB code: 6DBN), the common moiety with nitrogen, in-housed moiety #21, while in-housed moiety #6, forms a hydrogen bond with hinge residue L959 (Fig. 4C). Similarly, in-house moiety #21 in the ligand HQB complexed with CLK1 (PDB code: 6Q8P) forms a hydrogen bond with hinge residue L244, and the Checkmol fingerprint #21 interacts with the DFG motif (Fig. 4D). In summary, eight common moieties play the important roles in ligands and could be critical for drug design.

Two identified specific moieties, specifically for one family out of 10 kinase families, were identified in the GSK and AKT families. The Checkmol moiety #41 was also verified in the complex of GSK3B with 34O (PDB code: 3F7Z) and formed a van der Waals force with the DFG motif (Fig. 4E). In addition, we utilized our model to predict 34O and discovered the contribution of the Shapley value. Interestingly, our model successfully predicted 34O as an inhibitor (IC_50_: 65 nM in BindingDB) and the moiety contribution of 34O indicated that the specific moiety Checkmol moiety #41 had the highest SHAP. On the other hand, the specific moiety Checkmol moiety #31 in the AKT family also could be found on the complex of AKT2 and the ligand G93 (PDB code: 3D0E and IC_50_ is 0.4 nM in BindingDB) (Fig. 4F). G93 also contains a common moiety, in-house moiety #20, and the specific moiety forms a hydrogen bond with the main chain of F294 in the DFG motif. Moreover, the specific moiety Checkmol moiety #31 showed the second contribution based on the Shapley value.

These results suggest that the common and specific moieties identified by our model were verified by the protein structure complexes and SHAP model. Furthermore, moieties play an important role in interacting with key motifs on the kinase and make a huge contribution as shown by the calculated SHAP.

## Conclusion and discussion

This study is the first to establish a RF prediction model for the kinase family. In the selected 10 kinase families, the prediction ability of the model was more than 80%. Furthermore, the moiety preference of kinase family inhibitors with important motifs (such as hinge, DFG motif, and P-loop) in the ATP pocket of protein kinase complexes was identified and validated. This research is helpful for the rapid screening of compounds suitable for experiments, and also provides some important moieties that can be considered when designing effective inhibitors. Finally, this study can help rapidly filter compounds and aid in drug discovery and design. In the future, we hope to improve black-box machine learning and discover more drug candidates.

## Methods

### Data sets

**Fig. 5:**
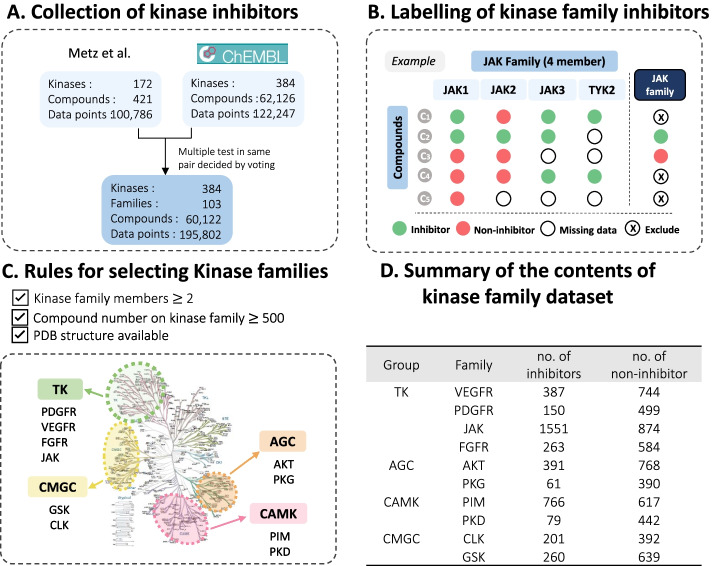
Strategies for processing and labeling of kinase family inhibitors. **A** Collection of kinase inhibitors.** B** Labelling of kinase family inhibitor.** C** Collected kinase family inhibitor data for RF model training and prediction.** D** Steps for filtering kinase family data set

To identify the data set of kinase family inhibitors, we first collected bioactivity data of kinase inhibitors from two data sets, for example, kinase profiling of Metz and ChEMBL database version 25. The former with 172 kinases, 421 compounds, and 100,786 data points in the form of *Ki* values, and only the data points with bioactivity data were considered. The latter was collected from ChEMBL and the assay which met the following criteria: (1) an IC_50_ value and (2) a confidence score of 9, was considered. This contained 384 kinases, 62,126 compounds, and 122,247 data points. To combine the two data sets, we converted the data points into binary labeling with the criteria *Ki* or IC_50_ < 1000 nM as active and *Ki* or IC_50_ > 1000 nM as non-active. To further prepare the robust dataset, when processing the multiple test results in the same protein kinase – compound pair, the labels were decided by voting. We collected a total of 384 kinases (including 103 kinase families); 60,122 compounds; and 195,802 data points (Fig. 5A).

Next, in order to achieve the predicted goal of kinase family inhibitors, we defined the criteria for whether the compound is a kinase family inhibitor and for those select kinase families that contain inhibitors that are enough in line with this research for modeling. To label a robust kinase family inhibitor (Fig. 5B), we annotated the following criteria: (1) compound *C*_*i*_ must be measured for more than half of the members of the target kinase family *f*_*j*_; (2) compound *C*_*i*_ must be in the same label type (active or non-active) for all the target kinase family *f*_j_, for example, *C*_*i*_ active for all the kinase family members is defined as a kinase family inhibitor, where *i* is the compound number of collected kinase compounds, and *j* is the number of kinases in the target kinase family. To ensure a predictive model with enough data to learn and explain interactions with protein complexes, we processed the kinase families with the following criteria: (1) the number of kinase members in the family ≥ 2; (2) dataset of kinase family inhibitor is sufficient for machine learning, the number of collected kinase family inhibitors ≥ 500; (3) kinase families were preserved when PDB complex structures (e.g., kinase bound with ligand) are available.

Finally, 10 kinase families were selected, including TK (PDGFR, VEGFR, JAK, FGFR families), CAMK (PIM, PKD families), CMGC (CLK, GSK families), and AGC (AKT, PKG families) groups. In total, 10,058 kinase inhibitors were collected. The detailed numbers of inhibitors or non-inhibitors are listed in Fig. 5D.

### Moieties representation

**Fig. 6:**
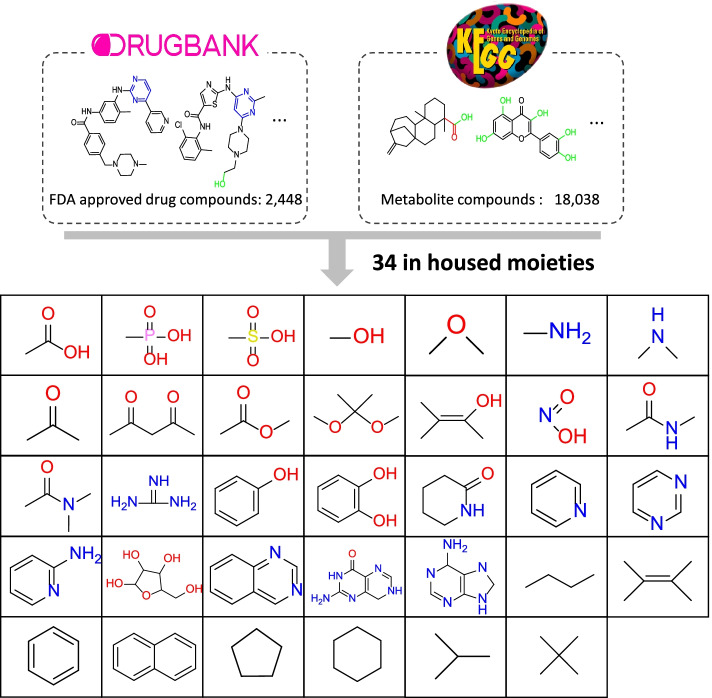
The list of the in-housed moieties collected from approved small molecule drugs from DRUGBANK and metabolites from KEGG

To build an interpretable model, the features of the machine learning method are key. Therefore, our work define “merged moiety-based interpretable features (MMIFs)” as features, which merged four moiety-based compound features, including Checkmol fingerprint [14], PubChem fingerprint, rings in drugs [15], and in-housed moieties. The Checkmol fingerprint includes 204 molecular fingerprint descriptions, including oxygen, nitrogen, sulfur, and other atoms with different bonds of surrounding atoms and different small substructures on small molecules. However, the Checkmol fingerprint lacks diverse rings (i.e., only aromatic and heterocyclic rings). Consequently, we added ring moieties from two resources: the PubChem fingerprint, which consists of 168 molecular fingerprints, including 4–6 carbon rings and resonance rings connected with carbon, oxygen, nitrogen, chlorine, or sulfur atoms. The PubChem fingerprint considers only pure carbon rings. The other ring source is from rings in drugs, and 139 ring structures commonly appear in drugs. Moreover, to improve the moiety inheritability, we generated in-housed moieties from metabolites and approved small-molecule drugs. We collected 18,028 metabolites from the KEGG compound database [17] and 2448 approved small-molecule drugs from the DRUGBANK database [18] to generate fragments from the pipeline pilot. Over 3,000 moieties were generated, and the top moieties were pre-defined as in-housed moieties by statistical analysis of the frequency of each moiety. Finally, we chose the top 34 common moieties as our in-housed features, as shown in Fig. 6. Finally, the MMIFs combine three public fingerprints and housed moieties. After removing duplicate moieties, a total of 518 moieties were considered as MMIFs. The kinase family inhibitors were converted into a binary sequence of MMIFs as input for the RF model.

To evaluate the performance and interpretability of different features, we also trained the model with widely used features, the extended-connectivity fingerprint of diameter 4 (ECFP4) [19], which encoded presence or absence by layered atom environments accounting for molecular topology using a hash function, and MACCS [20], encoded by 161 predefined substructures that are frequently used as molecular representations [10, 21–23].

### Kinase family classification RF model

The RF methodology was used on the MMIFs to create a kinase family inhibitor binary prediction model for each kinase family. The RF architecture proposed by Breiman in 2001 [24] is currently the most popular ensemble in machine learning.

In our study, we used an RF classifier from Scikit-learn of Python [25]. General parameters were set and tested, including the number of estimators (decision trees) being twofold from 50 to 2,000, maximum number of features (consider how many features on split each node): sqrt or log2 of all feature numbers, max depth as none, 20, 30, …, 100, min samples split as 2, 5, 10, bootstrap method as true (used) and false, and the class weight as none, balanced subsample, or balanced. Through hyperparameter grid searches on 1,296 combinations, the best hyperparameter was selected based on the cross-validation accuracy for each kinase family model. After hyperparameter grid searches, we calculated the average performance by tenfold cross validation and decided parameters: the number of estimators used was 1000, the maximum number of features was log2 of all feature numbers, the bootstrap method was used, class weight was balanced, and other hyperparameters used default values from scikit-learn.

For each kinase family predictive classification model, compounds were divided into 80% training and 20% test data, and model performance was estimated on the test set using accuracy, sensitivity (true positive), specificity (true negative), and Matthew’s correlation coefficient (MCC) [26]. The results of the performance indexes of the model are summarized in Table 1.

**Table 1:** Summary of the performance tables and collected data sets for 10 kinase families

Group	Family	Accuracy	Sensitivity	Specificity	MCC	No. of inhibitors	No. of non-inhibitor
TK	VEGFR	0.86 ± 0.06	0.73 ± 0.12	0.93 ± 0.06	0.68 ± 0.16	387	744
	PDGFR	0.92 ± 0.06	0.75 ± 0.22	0.94 ± 0.02	0.78 ± 0.17	150	499
	JAK	0.94 ± 0.02	0.95 ± 0.05	0.91 ± 0.03	0.86 ± 0.07	1551	874
	FGFR	0.9 ± 0.06	0.76 ± 0.13	0.93 ± 0.04	0.75 ± 0.13	263	584
AGC	AKT	0.94 ± 0.03	0.94 ± 0.06	0.95 ± 0.04	0.87 ± 0.08	391	768
	PKG	0.92 ± 0.07	0.62 ± 0.08	0.96 ± 0.02	0.6 ± 0.25	61	390
CAMK	PIM	0.92 ± 0.03	0.92 ± 0.06	0.91 ± 0.06	0.84 ± 0.07	766	617
	PKD	0.86 ± 0.09	0.56 ± 0.07	0.92 ± 0.04	0.63 ± 0.11	79	442
CMGC	CLK	0.8 ± 0.08	0.63 ± 0.08	0.89 ± 0.1	0.57 ± 0.06	201	392
	GSK	0.81 ± 0.07	0.57 ± 0.04	0.91 ± 0.05	0.59 ± 0.07	260	639

### Shapley value for identification of important moieties

The concept of the Shapely value (SHAP) was developed to estimate the importance of an individual player in a collaborative team, and distributed the total gain between players depending on the contributions of the final outcome of a game [27]. Currently, the SHAP provides a new solution for estimating the feature importance of applying a machine learning explanation. Therefore, in our study, we utilized the SHAP to open the black box of the RF model. The Python library SHAP was used to obtain the Shapley value of the predicted inhibitors.

### Identification of common and specific moieties

To infer common and specific moieties, we calculated common and specific scores. First, we created a union set of the top 30 important moieties of all families, and 69 moieties became candidate moieties. Then, we normalized the absolute SHAP value for each family to 0–1. Third, to compare the degree of importance across families, we calculated the average ($\mathrm{AvgNSHAP}$) and standard deviation value ($\mathrm{StdNSHAP}$) of normalized SHAP across families. $\mathrm{AvgNSHAP}$ and $\mathrm{StdNSHAP}$ are defined as follows:

$${\mathrm{AvgNSHAP}}_{i}=\frac{{\sum }_{1}^{NF}{\mathrm{NSHAP}}_{i}}{NF}$$

$${\mathrm{StdNSHAP}}_{i}=\sqrt{\frac{1}{NF}{\sum }_{i=1}^{NF}{\left({\mathrm{NSHAP}}_{i}-{u}_{i}\right)}^{2}}$$

where NF is the number of families, ${\mathrm{NSHAP}}_{i}$ is the normalized SHAP of moiety *i*, and ${u}_{i}$ is the average of the SHAP.

Next, we identified the common and specific moieties criteria as follows: For common moieties, the moiety should be of the same degree of importance across all families and therefore satisfy criteria (1) and (2). Therefore, we define two criteria: (1) and (2).

1 $${AvgNSHAP}_{ i}\ge third~quartile~of~ {AvgNSHAP}_{all~moieties}$$

2 $${StdNSHAP}_{i}\ge Average ~of~ {StdNSHAP}_{all~moieties}$$

Criteria (1) means that moiety $i$ should be greater than the third quartile (Q3) of ${{AvgNSHAP}_{all~ moiety}}$ and criteria (2) means moiety $i$ on all families should have a similar degree of importance. Finally, eight common moieties were identified.

To identify specific moieties, moiety $i$ should be distinguished across families, for example, only important in a specific family. We set criteria (3) and (4) to show the maximum on ${NSHAP}_{ i}$ to be significant.

3 $$\frac{({Max}_{1st}{NSHAP}_{i}^{NF}- {AvgNSHAP}_{i})}{{AvgNSHAP}_{i}} \ge 7$$

4 $$\frac{{(Max}_{2nd}{NSHAP}_{i}^{NF}- {AvgNSHAP}_{i})}{{AvgNSHAP}_{i}} \ge 7$$

where ${Max}_{1st}{NSHAP}_{i}^{NF}$ is the 1st significant value of moiety *i* across the families, and ${Max}_{2nd}{NSHAP}_{i}^{NF}$ is the 2nd significant value of moiety *i* across the families. Criteria (3) and (4) were greater than 7 were statistically analyzed and two specific moieties were identified.

## Data Availability

The datasets generated during and/or analyzed during the current study are available in the ChEMBL database [13, 28], https://www.ebi.ac.uk/chembl/.

## Abbreviations

FDA: Food and Drug Administration
MMIFs: Merged moiety-based interpretable features
RF: Random forest
SHAP: Shapley Additive exPlanations
PDB: Protein data bank
